# Supplementary material for: Enhanced Succinate Oxidation with Mitochondrial Complex II Reactive Oxygen Species Generation in Human Prostate Cancer
Source: Int J Mol Sci. 2022 Oct 12;23(20):12168. doi: 10.3390/ijms232012168 (PMC9603078; doi:10.3390/ijms232012168)
Supplement: Supplementary file 1 [file ijms-23-12168-s001.zip › ijms-1945829-supplementary.pdf]

Table S1. Patient demographics and prostate biopsy characteristics.

**Patients**	
Total	27
Age (years)	
Average (mean ± SD)	64.74 ± 6.87
Range	53 - 76
**Prostate gland**	
Total glands procured for biopsy	23
Gleason Score [1]	
3+3, 3+4	9
4+3, 4+4, 4+5	14
**Biopsy**	
Total biopsies [2]	96
3+X non-malignant	27
3+X malignant	13
4+X non-malignant	40
4+X malignant	16
Percentage of malignant biopsies/gland	
< 25%	10
≥ 26% and <50%	3
≥ 50% and <75%	7
≥ 75%	3

[1] clinical post-operative Gleason Score. [2] individual experimental biopsy Gleason Score.
